# Blockade of Autocrine CCL5 Responses Inhibits Zika Virus Persistence and Spread in Human Brain Microvascular Endothelial Cells

**DOI:** 10.1128/mBio.01962-21

**Published:** 2021-08-17

**Authors:** Megan C. Mladinich, Jonas N. Conde, William R. Schutt, Sook-Young Sohn, Erich R. Mackow

**Affiliations:** a Department of Microbiology and Immunology, Stony Brook University, Stony Brook, New York, USA; b Center for Infectious Diseases, Stony Brook University, Stony Brook, New York, USA; c Molecular and Cell Biology Program, Stony Brook University, Stony Brook, New York, USA; Duke University School of Medicine

**Keywords:** CCL5, ERK1/2, Zika virus, endothelial cell, persistence, survival, therapeutic

## Abstract

Zika virus (ZIKV) is a neurovirulent flavivirus that uniquely causes fetal microcephaly, is sexually transmitted, and persists in patients for up to 6 months. ZIKV persistently infects human brain microvascular endothelial cells (hBMECs) that form the blood-brain barrier (BBB) and enables viral spread to neuronal compartments. We found that CCL5, a chemokine with prosurvival effects on immune cells, was highly secreted by ZIKV-infected hBMECs. Although roles for CCL5 in endothelial cell (EC) survival remain unknown, the presence of the CCL5 receptors CCR3 and CCR5 on ECs suggested that CCL5 could promote ZIKV persistence in hBMECs. We found that exogenous CCL5 induced extracellular signal-regulated kinase 1/2 (ERK1/2) phosphorylation in hBMECs and that ERK1/2 cell survival signaling was similarly activated by ZIKV infection. Neutralizing antibodies to CCL5, CCR3, or CCR5 inhibited persistent ZIKV infection of hBMECs. While knockout (KO) of CCL5 failed to prevent ZIKV infection of hBMECs, at 3 days postinfection (dpi), we observed a >90% reduction in ZIKV-infected CCL5-KO hBMECs and a multilog reduction in ZIKV titers. In contrast, the addition of CCL5 to CCL5-KO hBMECs dose-dependently rescued ZIKV persistence in hBMECs. Inhibiting CCL5 responses using CCR3 (UCB35625) and CCR5 (maraviroc) receptor antagonists reduced the number of ZIKV-infected hBMECs and ZIKV titers (50% inhibitory concentrations [IC_50_s] of 2.5 to 12 μM), without cytotoxicity (50% cytotoxic concentration [CC_50_] of >80 μM). These findings demonstrate that ZIKV-induced CCL5 directs autocrine CCR3/CCR5 activation of ERK1/2 survival responses that are required for ZIKV to persistently infect hBMECs. Our results establish roles for CCL5 in ZIKV persistence and suggest the potential for CCL5 receptor antagonists to therapeutically inhibit ZIKV spread and neurovirulence.

## INTRODUCTION

Zika virus (ZIKV) is a mosquito-borne flavivirus (FV) associated with encephalitis, Guillain-Barré syndrome, and outbreaks of *in utero* fetal microcephaly (1–4). ZIKV crosses the blood-brain barrier (BBB) and damages the central nervous system (CNS) by lytically infecting neurons, neural progenitors, and astrocytes (5–7). In contrast to other FVs, ZIKV is uniquely detected in bodily fluids for up to 6 months, crosses placental barriers, and is sexually transmitted (8–11). How ZIKV persists in patients after acute febrile illness remains an enigma key to clearing ZIKV from patients and preventing ZIKV spread. In contrast to lytic infection of neurons, we reported that ZIKV persistently and nonlytically infects primary human brain microvascular endothelial cells (hBMECs) *in vitro* (12). We found that ZIKV is persistently released from basolateral and apical surfaces of polarized hBMECs without altering monolayer permeability (12). ZIKV persistence in hBMECs suggests a viral reservoir for systemic spread and a direct pathway for ZIKV to enter neuronal compartments.

The endothelium functions as a barrier that restricts viral entry into protected compartments (11, 13, 14). In the brain, a network of microvascular endothelial cells (ECs) in contact with basolateral astrocytes and pericytes forms the BBB that protects the CNS from viruses, immune cells, and circulating factors (15, 16). The critical nature of the BBB in restricting viral entry into neuronal compartments is evident from findings that intracranial inoculation of nonneurovirulent viruses results in lethal CNS damage (17). ZIKV persistence in hBMECs and basolateral spread provide a mechanism for ZIKV to cross the BBB and gain access to neurons.

*In vitro*, ZIKV persistently and productively infects and spreads in hBMECs without cytopathic effects for >9 days and following hBMEC passage (12). This is in contrast to lytic ZIKV infection of Vero E6 cells, neurons, and neuronal progenitors (5, 18, 19). ECs are unique cell types that dynamically secrete and respond to vascular growth factors and immunological cues that regulate antiapoptotic and proliferative responses to maintain vascular barrier functions (20–22). Interferon (IFN) was previously shown to foster EC survival (23–25); however, IFNs are not secreted from ZIKV-infected hBMECs despite their induction (12). The absence of secreted IFN is not the result of ZIKV-restricted secretion, as CCL5 is highly induced and secreted by ZIKV-infected hBMECs (12). It remains unknown how ZIKV posttranscriptionally regulates IFN-β/γ secretion and nonlytically persists within hBMECs.

CCL5/RANTES is a CC chemokine that recruits immune cells to inflammatory sites with specific effects on NK and T cells (26, 27). CCL5 is expressed by several cell types, including T cells, macrophages, eosinophils, and endothelial cells (26, 27). CCL5 activation is complex; however, IFN, interleukin-1 (IL-1), and tumor necrosis factor alpha (TNF-α) induce CCL5 via transcription factors, including NF-κB, interferon regulatory factor 1 (IRF1), IRF3, IRF7, or STAT1 (28, 29). CCL5 binds G-protein-coupled receptors (GPCRs), CCR1, CCR3, or CCR5, resulting in cell type-specific signaling responses (28, 30, 31). In the brain, CCL5 facilitates astrocyte proliferation via phosphatidylinositol 3-kinase (PI3K) and mitogen-activated protein kinase (MAPK) signaling pathways and supports neuronal function (32). The CCL5-CCR axis is also associated with tumor growth, metastasis, and angiogenesis in pancreatic, lung, and breast cancer (33–35). In macrophages, CCL5-CCR5 signaling directs antiapoptotic PI3K-AKT or MEK-extracellular signal-regulated kinase (ERK) cell survival programs that direct cell proliferation (36).

ECs secrete CCL5 and constitutively express CCL5 receptors (CCR3/CCR5) on their surfaces; however, the effects of CCL5 on hBMEC responses remain enigmatic (37). Secreted CCL5 reportedly forms a filamentous complex on the cell surface of ECs that directs the chemotaxis of immune cells to the endothelium (38). The activation of the PI3K-AKT and MEK-ERK pathways reportedly protects ECs against apoptosis (39–44), yet roles for autocrine CCL5 in hBMEC signaling and cell survival remain largely unknown. Given CCL5’s role as an immune and cancer cell survival factor, we hypothesized that CCL5 secreted by ZIKV-infected hBMECs could similarly promote prosurvival CCR3/CCR5 signaling responses that permit ZIKV to persistently infect hBMECs.

In this report, we determined that CCL5-CCR3/5 responses promote the survival and persistence of ZIKV-infected hBMECs. Consistent with CCL5-directed responses, we found that CCL5 addition to hBMECs, or ZIKV-infected hBMECs, selectively enhanced ERK1/2 phosphorylation. Suggesting a role for CCL5 in ZIKV persistence, we found that antibody neutralization of CCL5/CCR3/CCR5 reduced the viability of ZIKV-infected hBMECs and the number of ZIKV-infected hBMECs at 3 days postinfection (dpi). To efficiently inhibit CCL5-directed hBMEC responses, we knocked out CCL5 in hBMECs. Wild-type (WT) hBMECs and CCL5 knockout (CCL5-KO) hBMECs were equally infected by ZIKV, but by 3 dpi, the viability of only ZIKV-infected CCL5-KO hBMECs was inhibited (>90%). Consistent with this, exogenous CCL5 addition to CCL5-KO hBMECs dose-dependently rescued ZIKV infection, hBMEC viability, and viral persistence. These findings strongly indicate that the survival of ZIKV-infected hBMECs is dependent on ZIKV-induced CCL5 and is required for ZIKV persistence in hBMECs.

Comparisons of ZIKV-infected CCL5-KO, CCR3-KO, and CCR5-KO hBMECs demonstrated that single CCR3 or CCR5 receptor KOs only partially reduced ZIKV persistence in hBMECs, while CCL5-KO efficiently inhibited ZIKV persistence, spread, and titers. In contrast, applying small-molecule CCR3 (UCB35625) and CCR5 (maraviroc) antagonists dose-dependently reduced ZIKV titers and ZIKV persistence in hBMECs, without cytotoxicity (45, 46). These findings suggest the potential of neutralizing CCL5 or using receptor antagonists to prevent or clear persistent ZIKV infections and therapeutically inhibit ZIKV transmission to neuronal compartments and across placental barriers.

## RESULTS

How RNA viruses establish persistent infections is a major question in virology and of central importance to the spread and recurrence of neurovirulent viruses. ZIKV persists in patients for up to 6 months and nonlytically infects primary hBMECs that form the BBB. ZIKV persistently infects hBMECs for >9 days, through serial passage of hBMECs, and is apically and basolaterally released from hBMECs (12). ZIKV persistence in hBMECs provides an extended period for ZIKV to spread systemically and basolaterally gain access to neuronal compartments. The mechanism by which ZIKV persistently infects hBMECs is fundamental to ZIKV pathogenesis.

### CCL5 directs ERK1/2 survival signaling responses in ZIKV-infected hBMECs.

**FIG 1:**
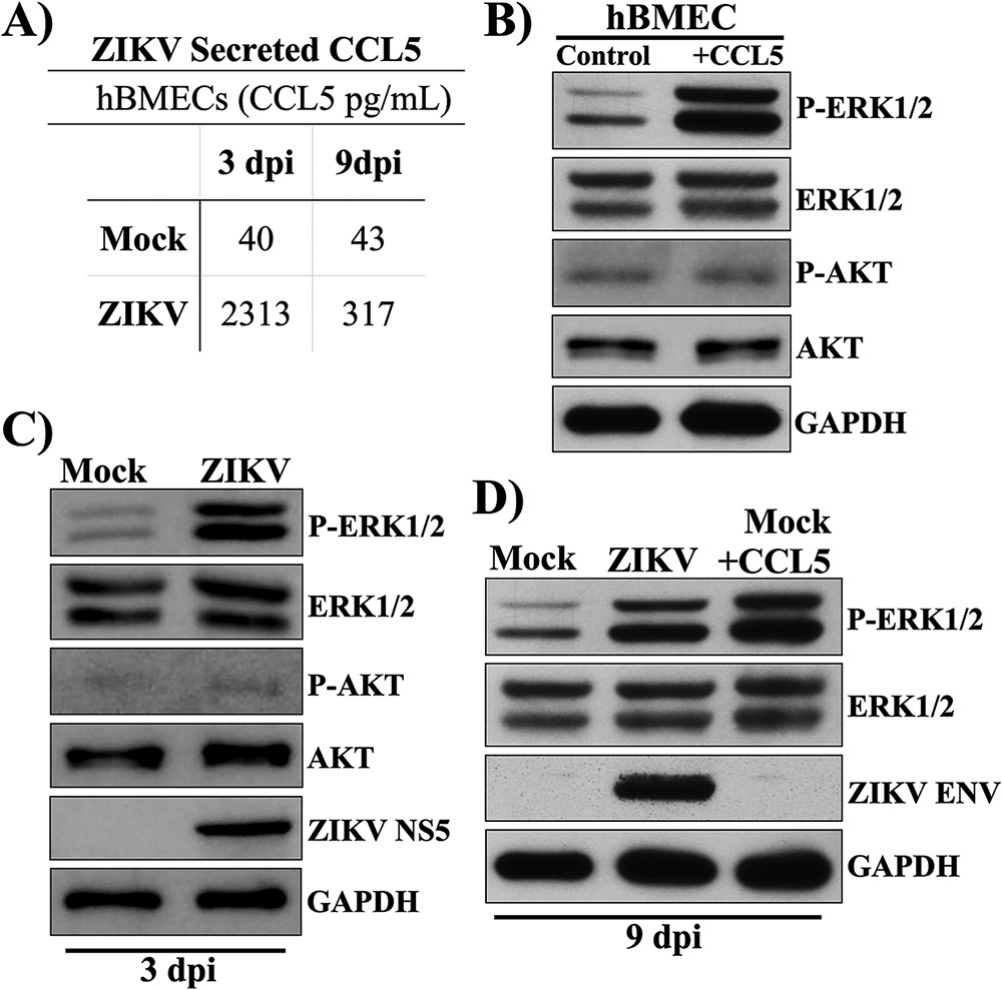
CCL5 directs ERK1/2 survival signaling responses in ZIKV-infected hBMECs. (A) Primary hBMECs were infected with ZIKV (PRVABC59) at an MOI of 10 or mock infected, and at 3 and 9 dpi, supernatants were analyzed by an ELISA (R&D Systems) for CCL5/RANTES relative to antigen standards. (B to D) hBMECs were starved overnight and CCL5 treated (100 ng/ml) (B) or mock or ZIKV infected at 3 and 9 dpi (C and D). Protein levels of ERK1/2, P-ERK1/2, AKT, P-AKT, and GAPDH controls were determined by Western blotting. Asterisks indicate statistical significance (*, *P < *0.05; ***, *P* < 0.001). Experiments were performed at least 3 times with similar results.

The endothelium dynamically responds to growth factors and immunological cues that regulate antiapoptotic and proliferative responses (47). We previously reported that ZIKV infection highly induces CCL5 transcripts and CCL5 secretion from hBMECs (12) (Fig. 1A). CCL5 is a chemokine that directs antiapoptotic survival programs in immune cells (36); however, roles for CCL5 in hBMEC survival responses remain unresolved. To determine if hBMECs are responsive to exogenous CCL5, we treated hBMEC monolayers with CCL5 and examined cell survival pathway MEK-ERK1/2 or PI3K-AKT signaling responses. hBMECs treated with exogenous CCL5 increased ERK1/2 phosphorylation but had no effect on AKT phosphorylation (Fig. 1B). Consistent with high-level CCL5 secretion from ZIKV-infected hBMECs, we similarly observed an increase in phosphorylated ERK1/2, but not AKT, in ZIKV-infected hBMECs (Fig. 1C). In persistently infected hBMECs (9 dpi), we found that ERK1/2 phosphorylation was increased to levels comparable to those of ERK1/2 phosphorylation directed by exogenous CCL5 addition (Fig. 1D). These findings demonstrate that ERK1/2 is phosphorylated in response to CCL5 addition to hBMECs and that ZIKV infection of hBMECs similarly directs ERK1/2 phosphorylation. These results are consistent with secreted CCL5 from ZIKV-infected hBMECs directing the autocrine activation of ERK1/2 signaling responses that promote cell survival.

### CCL5-CCR3/5 neutralization decreases ZIKV infection in hBMECs.

**FIG 2:**
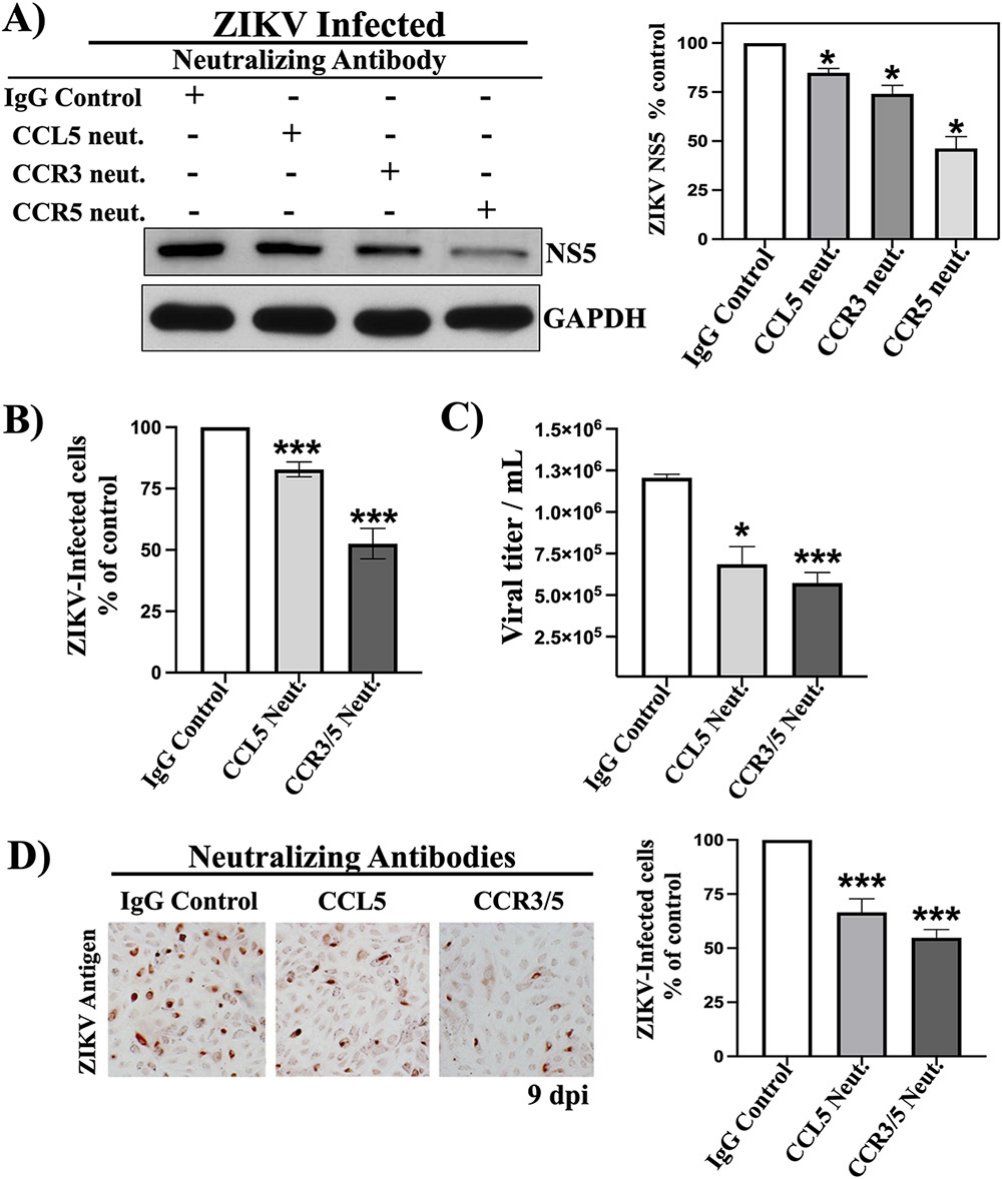
CCL5-CCR3/5 neutralization decreases ZIKV infection in hBMECs. hBMECs were infected with ZIKV (MOI of 5) and treated with isotype control or CCL5-, CCR3-, or CCR5-neutralizing antibodies (10× antibody ND50) added to supernatants once daily. (A) hBMECs were starved overnight at 3 dpi, and ZIKV NS5 was quantified by Western blot analysis and normalized to GAPDH levels. (B) ZIKV antigen-positive hBMECs were detected by anti-DENV4 hyperimmune mouse ascitic fluid (HMAF) and quantified at 3 dpi. (C) Viral titers of ZIKV-infected hBMEC supernatants were determined by a focus-forming unit (FFU) assay at 3 dpi. (D) At 9 dpi, daily neutralizing antibody-treated ZIKV-infected hBMECs were assessed for ZIKV antigen-positive hBMECs. Asterisks indicate statistical significance (*, *P < *0.05; ***, *P* < 0.001). Experiments were performed at least 3 times with similar results.

The CCL5 receptors CCR3 and CCR5 are expressed on hBMECs, and ZIKV-infected hBMECs highly induce and secrete CCL5 (37). CCL5 addition to hBMECs prior to or simultaneously with ZIKV adsorption had no effect on the initial ZIKV infection of hBMECs (see Fig. S1 in the supplemental material). To determine the effects of CCL5 on hBMECs, we first determined whether neutralizing antibodies to CCL5, CCR3, or CCR5 altered ZIKV infection and hBMEC viability. Neutralizing antibodies added to mock-infected hBMECs had no effect on cell viability compared to IgG controls (Fig. S2). The addition of CCL5- and CCR3-neutralizing antibodies resulted in small, ∼20% reductions in NS5 levels in ZIKV-infected hBMECs, while CCR5-neutralizing antibodies reduced NS5 levels by more than 50% compared to controls (Fig. 2A). Daily CCL5 neutralization decreased the number of ZIKV-infected hBMECs by only 20%, while neutralizing antibodies to CCR3/CCR5 receptors reduced ZIKV infection by 50% compared to controls (Fig. 2B). ZIKV titers were modestly reduced by 2-fold following CCL5 or CCR3/CCR5 antibody neutralization (Fig. 2C). The addition of CCL5- or CCR3/CCR5-neutralizing antibodies daily for 9 days reduced the number of ZIKV-infected hBMECs by 35% and 45%, respectively, compared to IgG-treated controls (Fig. 2D). Although CCL5 neutralization failed to dramatically reduce ZIKV-infected cells, NS5 protein expression, or viral titers, we questioned whether daily antibody additions were sufficient to neutralize constitutive high-level CCL5 secretion from ZIKV-infected hBMECs.

10.1128/mBio.01962-21.1 FIG S1 CCL5 does not affect ZIKV infection of hBMECs. hBMECs were pretreated with CCL5 (100 ng/ml) for 1 h followed by ZIKV infection (MOI of 10) or the simultaneous addition of ZIKV and CCL5. At 1 dpi, hBMECs were quantified for ZIKV antigen by immunoperoxidase staining. Download FIG S1, TIF file, 0.06 MB. Copyright © 2021 Mladinich et al. 2021 Mladinich et al. https://creativecommons.org/licenses/by/4.0/ This content is distributed under the terms of the Creative Commons Attribution 4.0 International license.

10.1128/mBio.01962-21.2 FIG S2 CCL5-CCR3/5 neutralization does not affect hBMEC viability. hBMECs were treated with IgG control or CCL5-, CCR3-, or CCR5-neutralizing antibodies twice daily for 3 days. Monolayers were assessed for cell viability via calcein-AM (green [live cells])/propidium iodide (red [dead cells]). Download FIG S2, TIF file, 0.2 MB. Copyright © 2021 Mladinich et al. 2021 Mladinich et al. https://creativecommons.org/licenses/by/4.0/ This content is distributed under the terms of the Creative Commons Attribution 4.0 International license.

### CCL5-KO reduces hBMEC viability during ZIKV infection.

**FIG 3:**
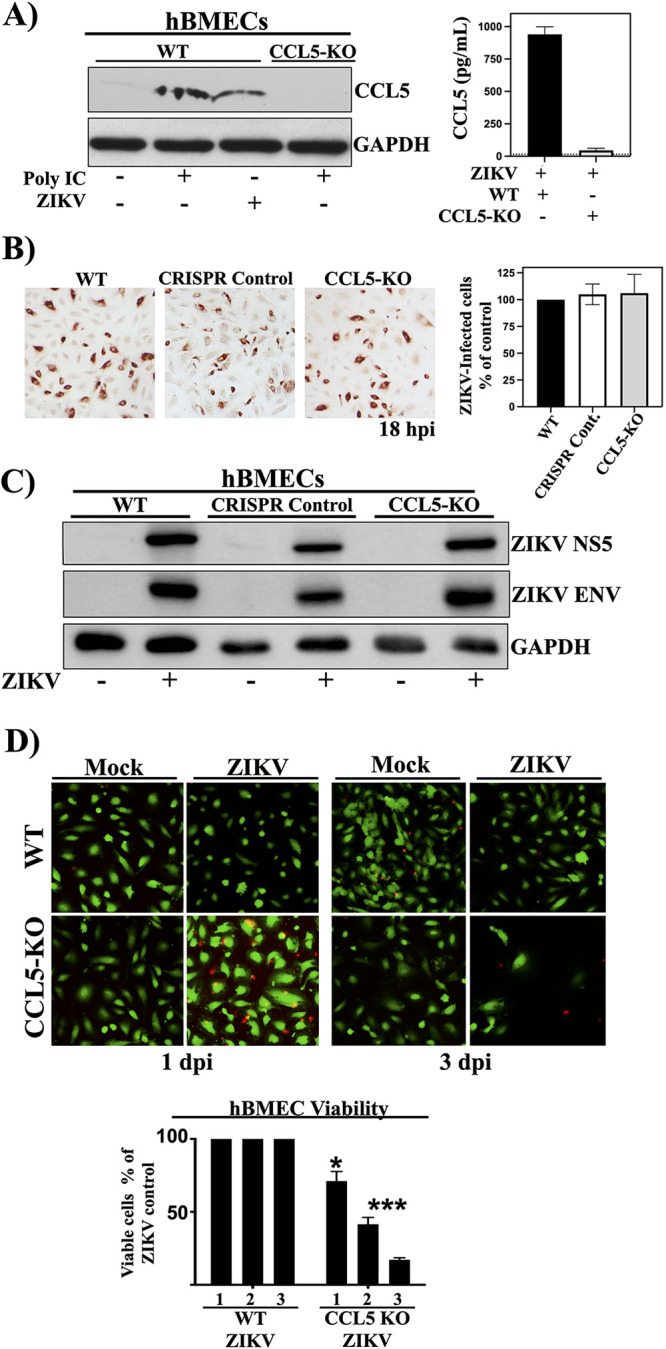
CCL5-KO reduces hBMEC viability during ZIKV infection. (A) WT and puromycin-selected CCL5-KO hBMECs were transfected with poly(I/C) (0.5 μg/ml) or ZIKV infected (MOI of 10) and evaluated for CCL5 expression by Western blotting and an ELISA (24 h posttransfection). (B and C) WT, CRISPR control, and CCL5-KO hBMECs were ZIKV infected (MOI of 10), and at 18 hpi, comparable ZIKV infections were detected by ZIKV antigen-positive cells (B) and Western blot detection of ZIKV NS5 and ZIKV Env, compared with GAPDH controls (C). (D) hBMECs were costained with calcein-AM (live)/propidium iodide (dead), and the viability of hBMECs was quantified by CyQuant analysis at 1 to 3 dpi compared to mock-infected WT hBMECs. Experiments were performed at least 3 times with similar results.

To bypass the limitations of antibody neutralization, we used a knockout approach to analyze the effects of CCL5 on ZIKV persistence. hBMECs were transduced with control or CCL5-targeting CRISPR-Cas9 knockout lentiviruses and puromycin selected. CCL5-KO hBMECs were CCL5 deficient by an enzyme-linked immunosorbent assay (ELISA) and Western blotting (WB) following poly(I/C) induction (Fig. 3A). ZIKV comparably infected WT, CRISPR control, and CCL5-KO hBMECs (18 h postinfection [hpi]), as determined by quantifying ZIKV antigen-positive cells (Fig. 3B) and NS5 protein (Fig. 3C). Thus, knocking out CCL5 did not alter the ability of ZIKV to infect hBMECs or express viral proteins in hBMECs at early times after infection.

Knocking out CCL5 did not alter hBMEC viability, and WT hBMECs that were ZIKV infected were also nearly 100% viable at 1 to 3 dpi (Fig. 3D). In contrast, ZIKV infection of CCL5-KO hBMECs resulted in a 30% reduction in viable CCL5-KO hBMECs at 1 dpi and a dramatic 85% reduction in viable ZIKV-infected hBMECs at 3 dpi (Fig. 3D). These results indicate that CCL5 is critical to the survival of ZIKV-infected hBMECs and suggest that CCL5 is a potential target for preventing or resolving ZIKV persistence.

### Knockout of the CCL5 receptors CCR3 and CCR5 limits ZIKV infection of hBMECs.

**FIG 4:**
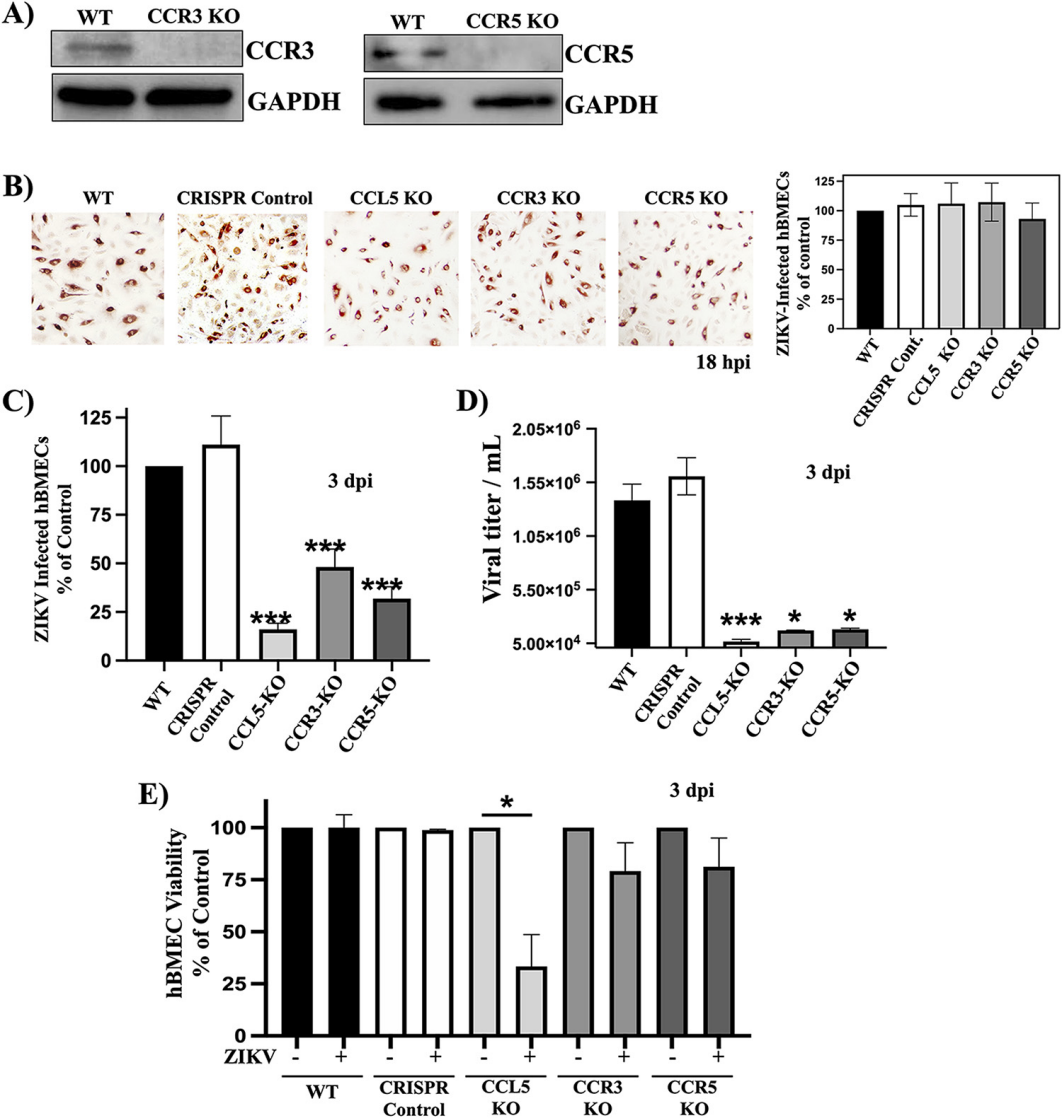
Knockout of the CCL5 receptors CCR3 and CCR5 limits ZIKV infection of hBMECs. (A) Puromycin-selected CCR3-KO and CCR5-KO hBMECs were evaluated for CCR3 or CCR5 protein expression, compared to GAPDH, by Western blotting. (B and C) WT, CRISPR control, and CCL5-KO hBMECs were ZIKV infected (MOI of 10), and ZIKV infection was detected by ZIKV antigen-positive cells at 18 hpi (B) and 3 dpi (C). (D and E) At 3 dpi, supernatants were assessed for viral titers (D), and viability was assessed via CyQuant uptake (E). Asterisks indicate statistical significance (*, *P < *0.05; ***, *P < *0.001). Experiments were performed at least 3 times with similar results.

CCL5 may signal through both the CCR3 and CCR5 receptors expressed on hBMECs to promote the survival of ZIKV-infected hBMECs. We generated CCR3 or CCR5 knockouts in primary hBMECs and confirmed CCR3-KO and CCR5-KO by WB after puromycin selection (Fig. 4A). ZIKV infected CCR3-KO or CCR5-KO hBMECs with efficiencies similar to those for WT hBMECs and CRISPR control hBMECs by staining of ZIKV-infected cells (18 hpi) (Fig. 4B). By 3 dpi, we found 50% and 35% reductions in the numbers of ZIKV-infected CCR3-KO and CCR5-KO hBMECs, respectively, and a 90% reduction in ZIKV-infected CCL5-KO hBMECs versus controls (Fig. 4C). Consistent with this, ZIKV titers that accumulated over 3 days were reduced by 1 to 1.5 logs in CCL5-KO, CCR3-KO, or CCR5-KO hBMECs (Fig. 4D). The viability of ZIKV-infected CCL5-KO hBMECs was reduced by 70%, whereas the viability of ZIKV-infected CCR3-KO and CCR5-KO hBMECs was reduced by ∼30% (Fig. 4E). These findings reveal that knocking out CCL5 inhibits ZIKV persistence in hBMECs by reducing the viability of ZIKV-infected cells. Although knocking out the CCR3/CCR5 receptors had less of an effect on ZIKV-infected cell viability, CCL5 may still act on the remaining CCL5 receptor present in individual receptor knockout hBMECs. Overall, these findings indicate that CCL5 plays an essential role in ZIKV-infected hBMEC survival and suggest that targeting CCL5 and CCL5 receptors has the potential to prevent ZIKV persistence and spread.

### Exogenous CCL5 rescues the viability and persistence of ZIKV-infected CCL5-KO hBMECs.

**FIG 5:**
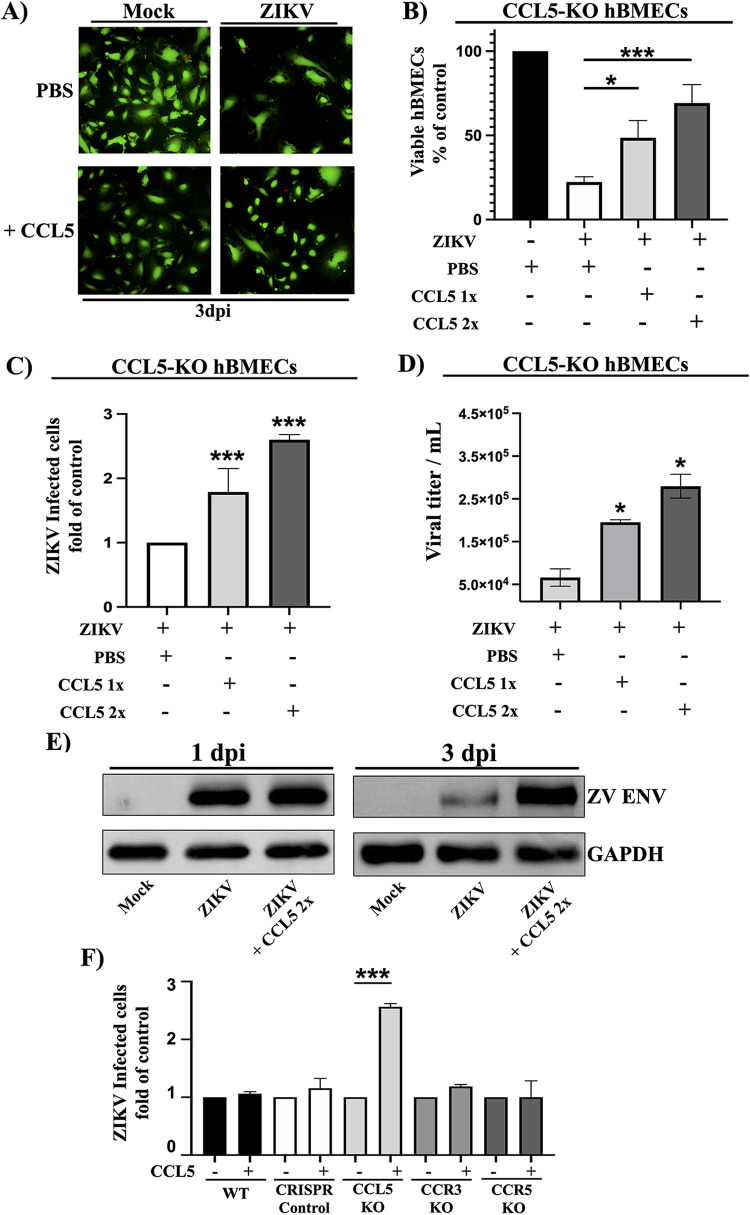
Exogenous CCL5 rescues the viability and persistence of ZIKV-infected CCL5-KO hBMECs. (A to D) CCL5-KO hBMECs were mock or ZIKV infected (MOI of 10) and control treated with PBS or CCL5 (100 ng/ml) once daily or twice daily to 3 dpi. At 3 dpi, CCL5-KO hBMECs were assessed for viability via calcein-AM/propidium iodide staining (A) and quantified by CyQuant analysis (B) for ZIKV-infected hBMECs (C) and viral titers (D). (E) Lysates of CCL5-KO hBMECs treated with PBS or CCL5 twice daily for 1 and 3 dpi were assessed for ZIKV (ZV) Env, compared to GAPDH controls. (F) WT, CRISPR control, CCL5-KO, CCR3-KO, and CCR5-KO hBMECs were ZIKV infected as described above; control treated with PBS or CCL5 (100 ng/ml) twice a day for 3 dpi; and assessed for ZIKV infection. Asterisks indicate statistical significance (*, *P < *0.05; ***, *P < *0.001). Experiments were performed at least 3 times with similar results.

To demonstrate that CCL5 directly affected the viability of ZIKV-infected hBMECs, we treated CCL5-KO hBMECs with exogenous CCL5 once or twice daily and assessed CCL5’s ability to rescue ZIKV persistence and replication in hBMECs. The viability of ZIKV-infected CCL5-KO hBMECs was restored by CCL5 addition and enhanced 2- to 5-fold by twice-daily CCL5 addition (Fig. 5B). Consistent with this, CCL5 addition to ZIKV-infected CCL5-KO hBMECs increased the number of ZIKV-infected cells (Fig. 5C), viral titers (Fig. 5D), and ZIKV Env protein expression (Fig. 5E). In contrast, CCL5 addition had no significant effect on the number of ZIKV-infected CCR3-KO or CCR5-KO hBMECs. These findings demonstrate that CCL5 rescues ZIKV persistence in CCL5-KO hBMECs, reveals the dependence of ZIKV persistence on the induction and secretion of CCL5, and demonstrates a prosurvival role of CCL5 in ZIKV-infected hBMECs that contributes to ZIKV persistence.

### Inhibiting the CCL5 receptors CCR3 and CCR5 restricts ZIKV persistence and spread in hBMECs.

**FIG 6:**
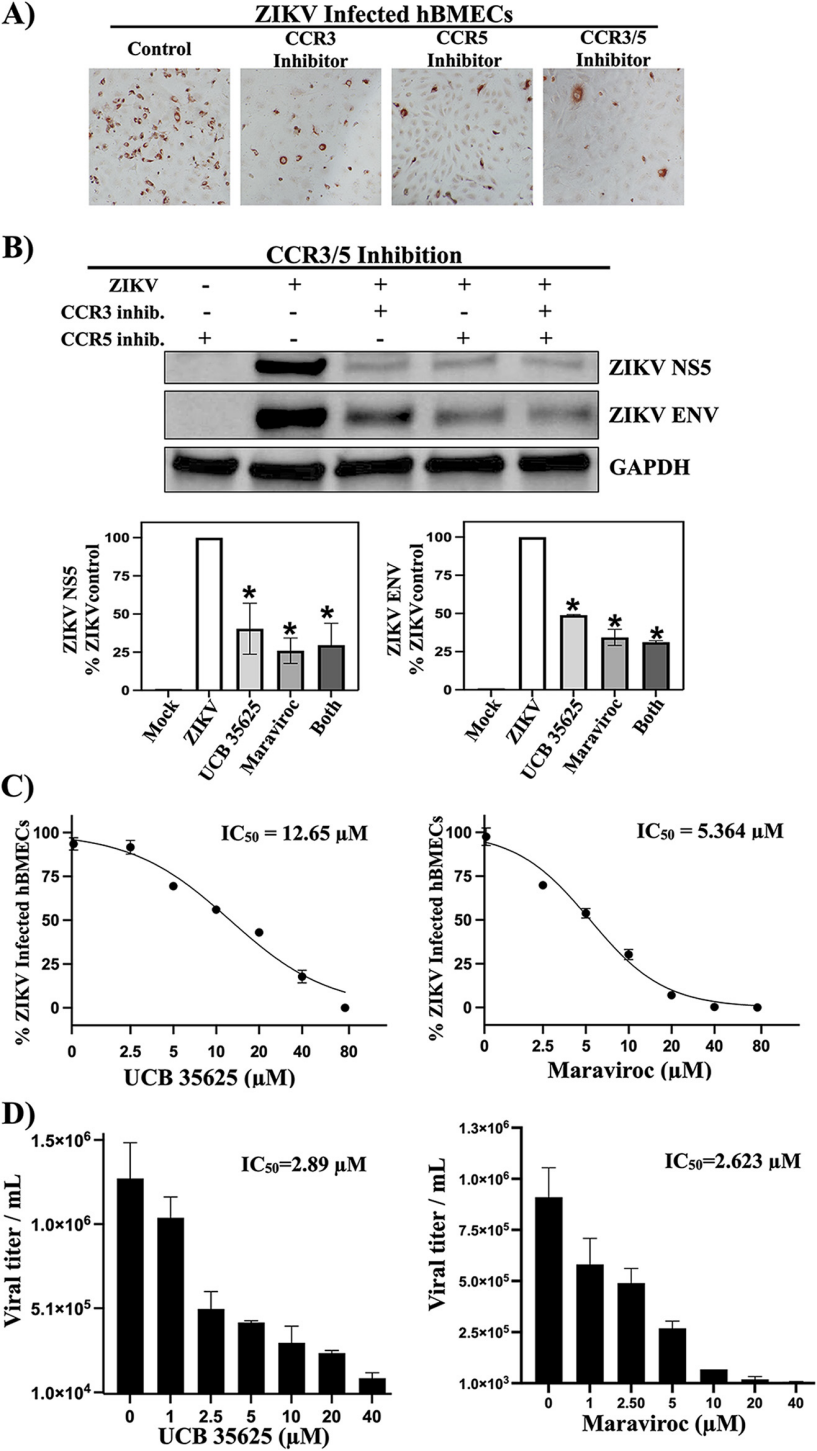
CCR3/CCR5 receptor antagonists restrict ZIKV persistence and viral titers in hBMECs. (A and B) hBMECs were ZIKV infected (MOI of 10) and treated with a CCR3 inhibitor (UCB35625) (20 μM), a CCR5 inhibitor (maraviroc; 20 μM), or both inhibitors twice daily to 3 dpi. The number of ZIKV-infected hBMECs was assessed at 3 dpi (A), and protein levels of ZIKV NS5 and ZIKV Env were quantified compared to GAPDH controls (B). (C and -D) Mock- and ZIKV-infected hBMECs were infected (MOI of 5) and treated with CCR3 or CCR5 inhibitors at 1 to 80 μM concentrations twice daily to 0 to 3 dpi. Treated hBMECs were quantified for the number of ZIKV-infected cells (C) and viral titers (D). Experiments were performed at least 3 times with similar results.

CCL5 receptors are therapeutically targeted by several nontoxic small-molecule inhibitors (aplaviroc, vicriviroc, maraviroc, GW766944, SB297006, and UCB35625) (45, 46, 48–51). In uninfected hBMECs, CCR3 (UCB35625) and CCR5 (maraviroc) antagonists, alone or together, inhibited CCL5-directed ERK1/2 activation (Fig. S3). Following ZIKV infection, CCR3 or CCR5 antagonists also dramatically reduced the number of ZIKV-infected hBMECs (Fig. 6A) and expressed NS5 and Env proteins (Fig. 6B). Dose-dependent analysis indicated that UCB35625 and maraviroc reduced the number of ZIKV-infected hBMECs and viral titers with 50% inhibitory concentrations (IC_50_s) of between 2.63 and 12.64 μM (Fig. 6C and D), without cytotoxicity (50% cytotoxic concentration [CC_50_] of >80 μM). These findings suggest the potential for CCL5 receptor antagonists to inhibit ZIKV persistence and spread in hBMECs.

10.1128/mBio.01962-21.3 FIG S3 Inhibition of the CCL5 receptors CCR3 and CCR5 inhibits CCL5-directed ERK1/2 activation of hBMECs. hBMECs were treated with a CCR3 inhibitor (UCB35625; 20 μM), a CCR5 inhibitor (maraviroc; 20 μM), or both inhibitors for 1 h prior to CCL5 stimulation (100 ng/ml). hBMEC lysates were assessed for protein levels of total ERK1/2 and phospho-ERK1/2, relative to GAPDH controls, by Western blotting. Download FIG S3, TIF file, 0.1 MB. Copyright © 2021 Mladinich et al. 2021 Mladinich et al. https://creativecommons.org/licenses/by/4.0/ This content is distributed under the terms of the Creative Commons Attribution 4.0 International license.

## DISCUSSION

Zika virus uniquely persists in patients for up to 6 months, is sexually transmitted, and is associated with encephalitis, neuronal demise, and microcephaly (5, 7, 8, 11). Our previous findings demonstrate that ZIKV persistently infects hBMECs (12), suggesting a cellular niche that fosters prolonged ZIKV replication, systemic spread, and entry into neuronal compartments. How ZIKV establishes persistent infection, evades innate and inflammatory immune responses, and induces cell survival responses remains a question that is central to ZIKV neuropathogenesis. ZIKV productively infects hBMECs without cytopathology, and ZIKV-infected hBMECs elicit an array of responses with the potential to impact cell survival and viral persistence. Among these, we found CCL5 to be highly induced (2,300-fold) and secreted by ZIKV-infected hBMECs (12). CCL5 is a well-studied chemokine that recruits, activates, and directs leukocyte survival responses through CCR3/CCR5 receptors (26, 36, 52). Remarkably, hBMECs also express CCR3 and CCR5, yet roles for CCL5 in regulating hBMEC responses remain largely unknown (37). Our findings suggest the potential for ZIKV-induced CCL5 to promote hBMEC survival and prompted studies defining the role of CCL5 in ZIKV persistence in hBMECs.

The endothelium regulates capillary barrier functions that restrict viral and immune cell entry into protected neuronal compartments by dynamically responding to growth factors, chemokines, and cytokines (15, 16, 20, 53, 54). CCL5 is a determinant of proangiogenic inflammation that is largely ascribed to immune cell responses; however, selectins induce ECs to express CCL5, and filamentous aggregates of CCR3/CCR5 on ECs are suggested to promote immune cell attachment (38, 55). EC proliferation, angiogenesis, metastasis, and vascular stasis are also linked to the activation of ERK1/2 signaling responses and the role of this pathway in cell survival (43, 44, 56–58). ERK1/2 signaling directs vascular integrity by protecting ECs from receptor-mediated apoptosis and through EC proliferation in response to vascular endothelial growth factor (VEGF) (59). Despite this, there is little understanding of CCL5’s function in the autocrine activation of prosurvival ERK1/2 signaling responses in ECs.

Our findings demonstrate that the exogenous addition of CCL5 directs the phosphorylation of ERK1/2 in hBMECs, indicating that CCL5 activates a prosurvival signaling pathway in hBMECs. ZIKV highly induces CCL5 secretion from hBMECs, and consistent with this, we found that ERK1/2 is phosphorylated in ZIKV-infected hBMECs. To investigate a role for CCL5 in ZIKV persistence in hBMECs, we initially inhibited CCL5 or the CCR3/CCR5 receptors with neutralizing antibodies. Neutralizing antibodies to CCL5 resulted in a small but significant 25% reduction in the number of ZIKV-infected hBMECs. CCL5 antibodies were likely to have been insufficient to neutralize the high level of ZIKV-induced CCL5 and its autocrine effects. Antibody blockade of CCR3 and CCR5 receptors resulted in 20% and 50% reductions in ZIKV-infected hBMECs, respectively. These findings suggested divergent neutralizing CCR3 versus CCR5 efficacy and unique roles for CCR3 and CCR5 in ZIKV-infected hBMECs. However, CCR3 receptors are rapidly recycled after internalization, while CCR5 antibody-induced internalization degrades the CCR5-ERK1/2 signalosome (60), suggesting that the differences observed may instead be a consequence of discrete mechanisms of antibody-directed CCR3 and CCR5 receptor downregulation. We see modest effects of neutralizing antibodies on ZIKV infection, titers, and NS5 protein levels. However, this may not be an issue from a therapeutic perspective as the goal is the elimination of persistently infected cells rather than the arrest of acute ZIKV replication.

To prevent highly expressed CCL5 from escaping antibody neutralization, we evaluated the effect of blocking ZIKV-induced CCL5 using CCL5-KO hBMECs. Although ZIKV comparably infected WT and CCL5-KO hBMECs (Fig. 4B), the number of ZIKV-infected CCL5-KO hBMECs was reduced 90% by 3 dpi compared to WT hBMECs (Fig. 3D), with a dramatic reduction in the viability of ZIKV-infected CCL5-KO hBMECs. Like CCL5, single CCR3-KO or CCR5-KO hBMECs were found to significantly decrease the number of ZIKV-infected cells (40 to 50% of the WT) and ZIKV titers. However, the viability of ZIKV-infected hBMECs appears to be tempered by the presence of a second functional CCL5 receptor. Proving that the observed decrease in ZIKV-infected hBMECs was CCL5 specific, adding CCL5 exogenously to ZIKV-infected CCL5-KO hBMECs rescued the viability and number of ZIKV-infected CCL5-KO hBMECs and increased ZIKV titers in a dose-dependent manner (Fig. 5). These findings strongly suggest that, in the absence of CCL5, ZIKV is effectively cleared from hBMECs and that CCL5 is an essential factor required for ZIKV persistence in hBMECs.

The roles of CCL5 in angiogenic repair and protection of EC integrity are connected to CCL5’s role as a cell survival factor that promotes cell motility and metastasis (33, 61). The relationship between CCL5 expression and breast, colon, and prostate cancer is well established, and as a result, CCL5 and CCR5 have emerged as therapeutic targets for restricting metastatic cancer (33, 61). CCL5/CCR5 activation of ERK1/2 plays a prominent role in cancer cell survival and suggests an angiogenic mechanism for EC survival and barrier functions during persistent ZIKV infection (35, 61–66). Our findings suggest that high levels of CCL5 secretion from ZIKV-infected hBMECs direct the activation of prosurvival ERK1/2 signaling pathways and suggest further investigating the roles of ERK1/2 signaling in ZIKV persistence in hBMECs. However, ERK1/2 inhibition may be difficult to study in ECs as ERK1/2 inhibitors are cytotoxic to ECs (56), and the proliferation of ERK1/2 KO ECs is dramatically reduced *in vitro* (44). As CCL5-KO hBMECs are eliminated by ZIKV infection, and exogenous CCL5 addition restores the viability of ZIKV-infected CCL5-KO cells, our findings support a novel role for ZIKV-elicited CCL5 as a required survival factor that permits ZIKV to persist in hBMECs.

How RNA viruses establish persistent, long-term infections that extend beyond acute febrile illness and permit spread across normal placental, brain, and testicular barriers remains a fundamental question in virology with several requirements. RNA virus persistence necessitates viral regulation of innate and adaptive immune responses, a cellular niche that permits viral replication, viral endurance across cell division, and nonlytic viral infection of targeted cells (67, 68). The ability of ZIKV to persistently infect patients for up to 6 months indicates that ZIKV evades clearance by innate and adaptive immune responses and navigates a delicate balance of viral replication and host cell viability that differentiates ZIKV from other flaviviruses.

ZIKV uniquely regulates innate immune responses in hBMECs. ZIKV transcriptionally induces CCL5 as well as IFN-β and IFN-λ from infected hBMECs; however, only CCL5 is found to be secreted during infection. Consistent with this, added IFN-α/β blocks ZIKV infection of hBMECs, suggesting that if IFN-β were secreted by hBMECs, ZIKV spread and persistence in hBMECs would be completely inhibited (12, 69). Although hBMECs lack IFN-λ receptors and are unresponsive to IFN-λ, ZIKV regulation of IFN-λ secretion may play a central role in ZIKV’s ability to cross placental barriers. The mechanism by which ZIKV posttranscriptionally regulates IFN-β/λ secretion remains to be resolved and is another novel attribute that, like high-level CCL5 secretion, distinguishes ZIKV from other flaviviruses.

CCL5 is often referred to as a “double-edged sword” because it plays crucial roles in immune cell recruitment and activation that clear acute viral infections, yet CCL5 can also cause chronic inflammation that contributes to pathogenesis (26, 70, 71). Additional chemokines (CXCL10/11, CCL20, IL-1, and IL-6) are induced in ZIKV-infected hBMECs, which may similarly recruit and activate immune cells in the presence or absence of CCL5 (12). Despite these adaptive immune signals, ZIKV persistently infects patients, suggesting that chemokine responses fail to limit ZIKV infections to an acute febrile illness and permit or foster ZIKV persistence.

It remains unclear why CCL5 is highly induced in ZIKV-infected hBMECs in the absence of IFN induction and what signaling responses (i.e., NF-κB or IRFs) of hBMECs are uniquely engaged by ZIKV to highly induce CCL5. *In vivo*, high-level CCL5 expression may play an additional role in ZIKV escape from CD8^+^ T cell clearance. High-level CCL5 expression reportedly enhances regulatory T cell (T_reg_) cytotoxicity against CD8^+^ T cells, preemptively causing CD8^+^ T cell apoptosis that prevents the targeting and clearance of cancer cells (72). Yet CD8^+^ T cells play a protective role during ZIKV infection by reducing viral burdens in T cell-competent H-2^b^ mice, while the depletion of CD8^+^ T cells leads to higher mortality rates (73). Collectively, these findings suggest that high levels of CCL5 induced by ZIKV could suppress T cell clearance and provide a potential *in vivo* mechanism of ZIKV persistence. Roles for CCL5 in ZIKV-regulated CD8^+^ T cell responses are complicated in ZIKV-infected animal models and have yet to be addressed in ZIKV models with persistence or disease correlates.

CCL5 is reportedly induced by several FVs that cause acute febrile diseases but lack the ability to persistently infect cells or patients (74, 75). Dengue virus (DENV) infects ECs, induces low levels of CCL5 (84-fold for DENV versus 2,300-fold for ZIKV at 1 dpi), and fails to persistently infect ECs in part because DENV fails to inhibit IFN-β secretion by ECs (76). It is unclear whether CCL5 could direct DENV persistence in ECs if, like ZIKV, IFN-β responses were blocked. The unique high level of CCL5 expression distinguishes ZIKV infection of hBMECs and may play a key role in ZIKV persistence. In addition to novel CCL5 and IFN regulation by ZIKV, there are likely to be additional conditions, responses, and protein interactions that contribute to ZIKV’s persistence in specific cell types and patients that need to be resolved. Why ZIKV lytically infects neurons and IFN-deficient Vero E6 cells remains an enigma that is also tied to responses and virus regulation of discrete cell types (7, 8). This study reveals that CCL5 is required for ZIKV persistence in hBMECs and a potential viral clearance target.

CCL5 is suggested to contribute to viral entry into the CNS, inflammation, or T cell-directed neuroinflammatory damage induced by West Nile virus (WNV), Japanese encephalitis virus (JEV), rabies virus (RABV), tick-borne encephalitis virus (TBEV), measles virus (MV), and human cytomegalovirus (HCMV) (77–82). CCL5, CCR3, and CCR5 remain an association tied to a range of different inflammatory responses, the activation or regulation of CD8^+^ T cell responses, with suggested roles for viral entry into the CNS. However, FVs elicit many chemokine responses that factor into immunopathogenesis and FV neurovirulence independent of CCL5. The enhanced mortality of WNV-infected mice lacking the chemokine CXCL10 receptor CXCR3 demonstrates the requirement for CXCR3 in CD8^+^ T cell recruitment and WNV clearance from the CNS (83). Yet WNV and JEV pathogenesis is reportedly enhanced in CCR5-deficient mice, resulting in increased virus in the CNS and lethal disease (84–86). Contrary to this, treatment of mice with the CCR5 antagonist maraviroc reduced JEV-induced inflammation in the brain and increased the survival of JEV-infected mice (87). These disparate findings suggest the need for a more complete analysis of interconnected chemokine and immune cell responses, immune cell entry, and FV clearance from the CNS that contribute to disease.

Animal models of ZIKV persistence that permit assessing the roles for CCL5/CCR3/CCR5 in pathogenesis are lacking. ZIKV studies routinely use IFN-α receptor (IFNAR)-deficient mice as lethal animal models that succumb to high-level viral replication (88). However, unchecked viral replication in IFNAR-deficient mice lacks human disease correlates that reflect ZIKV persistence and spread. CCR3/5 inhibitors and CCL5- or CCR5-deficient mice are available for study. However, without ZIKV disease or persistence models, it remains unclear how ZIKV infection of IFNAR-deficient mice can be used to study interdependent CCL5 responses that impact immune cell targeting and viral persistence and spread to neuronal compartments.

CCR3 and CCR5 are redundant CCL5 receptors that appear to similarly activate cell signaling pathways (89). Since the discovery of CCR5 as an HIV coreceptor, there has been heightened interest in developing CCR5 antagonists, and several specific small-molecule antagonists (maraviroc, cenicriviroc, vicriviroc, TBR-652, and INCB9471) are now available and used for antiviral therapies (45, 90). A recent study of hepatitis C virus (HCV), a persistent flavivirus that induces CCL5, revealed that CCR5 blockade with maraviroc (clinically approved) or cenicriviroc inhibits HCV replication (91). Our findings reveal that the inhibitors maraviroc and UCB35625 restrict ZIKV infection and dose-dependently reduce ZIKV titers in hBMECs. These findings suggest that inhibitors targeting CCR3 and CCR5 contribute to ZIKV clearance from hBMECs. Treatment of ZIKV-infected hBMECs with UCB35625, maraviroc, or both inhibitors reduced ZIKV NS5 and Env protein levels but did not reveal if CCR3 and CCR5 have additive or synergistic functions on hBMECs. In the future, CCR3/5 double-receptor knockouts may need to be assessed to clarify the precise role of CCR3 and CCR5 in hBMECs and determine the most efficient way to therapeutically target the CCL5-CCR3/5 pathway to inhibit ZIKV persistence. However, these data overall demonstrate that CCL5-CCR3/5 signaling pathway responses play a critical role in ZIKV persistence in hBMECs and are potential therapeutic targets for resolving ZIKV persistence in patients.

ZIKV-infected hBMECs regulate a collection of inflammatory chemokine, IFN, and cell survival responses required for ZIKV persistence. Through a combination of CCL5-CCR3/5 neutralization, knockout, and receptor inhibition studies, we demonstrate that CCL5 signaling is critical for ZIKV persistence in hBMECs. Our findings establish that ZIKV-induced CCL5 acts in an autocrine manner to activate CCR3/5-directed ERK1/2 survival pathways, and consequently, ZIKV orchestrates CCL5 induction in order to elicit hBMEC responses required for the survival of ZIKV-infected hBMECs. Our data demonstrate that neutralizing antibodies and small-molecule CCR3/CCR5 receptor antagonists inhibit persistent ZIKV infection of hBMECs and as a result have the potential to prevent ZIKV spread to neuronal compartments and across placental barriers. These findings implicate CCL5, CCR3/CCR5, and ERK1/2 survival signaling pathways as potential therapeutic targets for clearing persistent ZIKV infections and preventing ZIKV spread and neurovirulence.

## MATERIALS AND METHODS

### Cells and virus.

C6/36 cells (ATCC CRL-1660) were grown in minimal essential medium (MEM) (10% fetal bovine serum [FBS], 1× nonessential amino acids [NEAA]) at 28°C in 5% CO_2_. Vero E6 cells (ATCC CRL 1586) and HEK293T cells (ATCC) were grown in Dulbecco’s modified Eagle’s medium (DMEM) (8% FBS) as previously described (12). Human brain microvascular ECs (passage 3) were purchased from Cell Biologics (catalog number H-6023), used at passages 4 to 10, and grown in Endothelial Cell Growth Basal Medium-2 (EBM-2) with SingleQuots (Lonza) at 37°C in 5% CO_2_. ZIKV (PRVABC59) was obtained from the ATCC and minimally passaged (multiplicity of infection [MOI] of 0.1 to 1) in C6/36 cells (MEM, 2% FBS). ZIKV titers were determined by a focus-forming assay in Vero E6 cells by immunoperoxidase staining with anti-DENV4 hyperimmune mouse ascitic fluid (HMAF) (ATCC) and 3-amino-9-ethylcarbazole (92, 93).

### Antibodies.

Anti-ZIKV envelope (catalog number GTX133314) and anti-ZIKV NS5 (catalog number GTX133312) were obtained from GeneTex; anti-ERK1/2 (catalog number 9102S), anti-phospho-ERK1/2 (P-ERK1/2) (catalog number 4370S), anti-AKT (catalog number 9272), and anti-phospho-AKT (catalog number 9271S) were obtained from Cell Signaling; anti-CCR3 (catalog number PA5-19859) and anti-CCR5 (catalog number PA5-78949) were obtained from Invitrogen; anti-CCL5 (catalog number K1014) and the IgG isotype control (VP16, catalog number F249) were obtained from Santa Cruz; and anti-glyceraldehyde-3-phosphate dehydrogenase (GAPDH) (catalog number G9545) was obtained from Sigma-Aldrich. Neutralizing antibodies to CCL5 (catalog number AF-278-NA), CCR3 (catalog number MAB155), CCR5 (catalog number MAB182), and CCL5/RANTES DuoSet ELISA kits were obtained from R&D Systems (12).

### Neutralization and inhibition.

hBMECs were treated with neutralizing antibodies or inhibitors every 12 h starting at 0 hpi and assayed at 3 dpi. Neutralizing antibodies were added to supernatants (10-fold the respective neutralization dose 50 [ND50]) to inhibit ∼0.3 ng/ml of CCL5 secreted by ZIKV-infected hBMECs. For neutralization experiments, mock- or ZIKV-infected hBMECs were treated once daily for 3 dpi with CCL5, CCR3, CCR5, or both CCR3 and CCR5 neutralizing antibodies or the IgG isotype control. For inhibition experiments, hBMECs were treated with dose dilutions (1 to 80 μM) of UCB35625 (Tocris), maraviroc (catalog number UK-427857; Selleckchem), or both inhibitors twice daily for 3 dpi.

### Lentiviral vectors.

The pLentiCRISPRv2 plasmid was purchased from Addgene (catalog number 52961). Prevalidated single guide RNA (sgRNA) from GenScript’s gRNA database for CCL5 (CACCGAGGTACCATGAAGGTCTCCG), CCR3 (CACCGCGCCTCTGCTCGTTA), CCR5 (TCAGTTTACACCCGATCCAC), and the CRISPR nontargeting control (GACGGAGGCTAAGCGTCGCAA) were cloned into pLentiCRISPRv2 (94). Lentivirus was generated in HEK293T cells by polyethylenimine (PEI) transfection (95). hBMEC transduction and puromycin selection (3 days; 0.5 μg/ml) were performed as previously described (69). Selected hBMEC lysates were analyzed by Western blotting for CCL5, CCR3, or CCR5 expression.

### CCL5 rescue.

hBMECs were ZIKV infected (MOI of 10) or mock infected, and the viability of infected cells and viral titers were assessed at 3 dpi. Alternatively, starved WT or transduced hBMECs were stimulated with 100 ng/ml CCL5 (catalog number 278-RN-050/CF; R&D Systems) every 12 h (0 to 72 hpi).

### Cell viability assays.

Live/dead and CyQuant viability assays were performed as previously described (12). For live/dead assays, hBMECs were costained with calcein-AM (live/green, 3 μM; Invitrogen) and propidium iodide (dead/red, 2.5 μM; Calbiochem). Calcein-AM-positive versus PI-positive cells were resolved using an Olympus IX51 microscope and overlaid using Adobe Photoshop. hBMECs were incubated with CyQuant-NF (Thermo Fisher), and fluorescence was quantified using a BioTek FLx800 fluorimeter.

### Western blotting.

Western blotting was performed as previously described (93). hBMECs were starved overnight and collected or stimulated with 100 ng/ml CCL5 for 10 min before harvest. Cells were washed with phosphate-buffered saline (PBS) and lysed in 1% NP-40 buffer with a protease inhibitor cocktail (Sigma) as previously described (12). Total protein levels were determined in a bicinchoninic acid assay (Thermo Fisher), and proteins were resolved by SDS–12% PAGE, transferred to nitrocellulose, blocked in PBS–1% bovine serum albumin (BSA), and incubated with antibodies in a blocker. Proteins were detected using horseradish peroxidase (HRP)-conjugated anti-mouse or anti-rabbit secondary antibodies (Amersham) and the Luminata Forte HRP substrate (Millipore).

### Statistical analysis.

The results shown in each figure were derived from 2 to 3 independent experiments with comparable findings; the data presented are means ± standard errors of the means (SEM), with the indicated *P* values of <0.01 and <0.001 considered significant. Two-way comparisons were performed by two-tailed analysis of variance and unpaired Student’s *t* test. All analyses were performed using GraphPad Prism 9.1.2.
