# Supplementary figures and images for: Blockade of Autocrine CCL5 Responses Inhibits Zika Virus Persistence and Spread in Human Brain Microvascular Endothelial Cells
Source: mBio. 2021 Aug 17;12(4):e01962-21. doi: 10.1128/mBio.01962-21 (PMC8406327; doi:10.1128/mBio.01962-21)

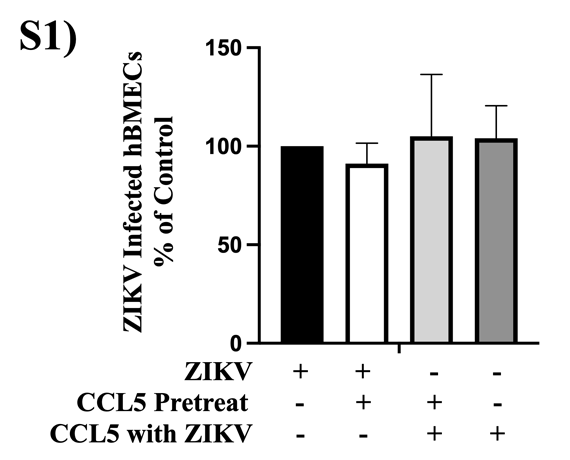

Supplement: FIG S1 [file mbio.01962-21-sf001.tif]

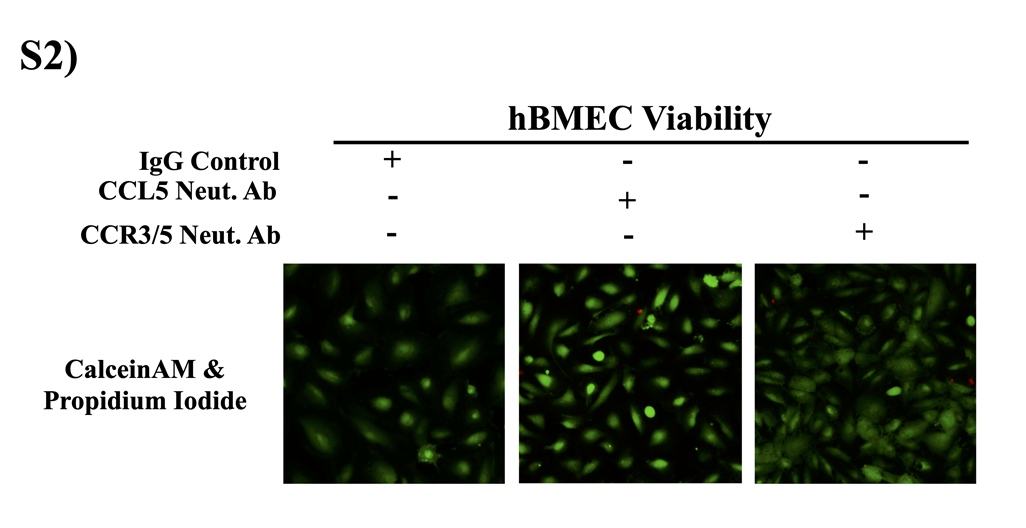

Supplement: FIG S2 [file mbio.01962-21-sf002.tif]

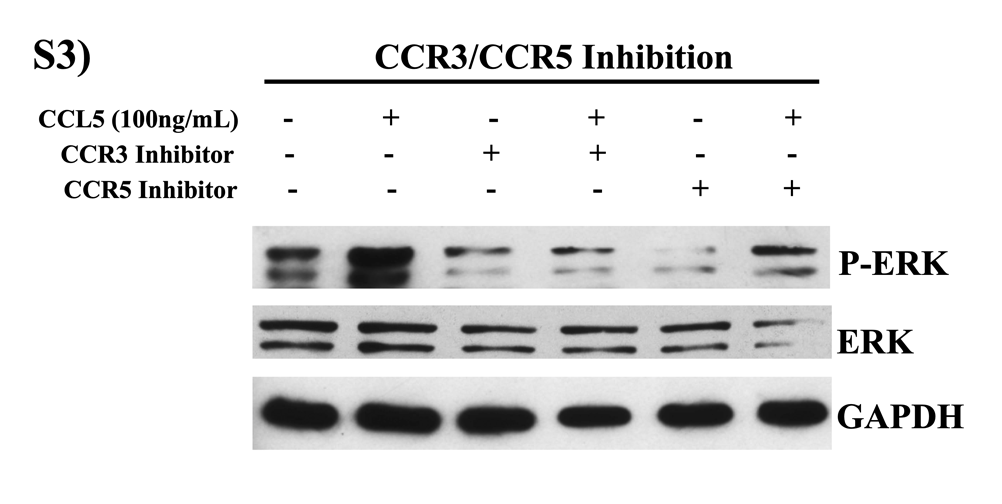

Supplement: FIG S3 [file mbio.01962-21-sf003.tif]
